# Rapid transcriptional reprogramming underlies Fusarium wilt resistance in strawberry: insights from comparative physiological and transcriptomic analyses

**DOI:** 10.3389/fpls.2026.1861706

**Published:** 2026-08-31

**Authors:** Hong Wan, Xiaoyuan Wang, Yanbing Wang, Na Xue, Jiwei Ruan, Deneng Liu, Zhiwei Zeng, Pang Tao, Qian Wang

**Affiliations:** 1 Horticulture Research Institute, Yunnan Academy of Agricultural Sciences, Kunming, Yunnan, China; 2 Engineering Research Center of Vegetable Germplasm Innovation and Support Production Technology of Yunnan Province, Kunming, Yunnan, China; 3 Faculty of Modern Agricultural Engineering, Kunming University of Science and Technology, Kunming, Yunnan, China; 4 Flower Research Institute, Yunnan Academy of Agricultural Sciences, Kunming, Yunnan, China; 5 Huize County Fruit Workstation, Huize, Yunnan, China; 6 Huize County Institute of Key Industrial Technology, Huize, Yunnan, China

**Keywords:** *Fusarium oxysporum* f. sp. *fragariae*, Fusarium wilt, resistance evaluation, strawberry, transcriptomic

## Abstract

**Introduction:**

Fusarium wilt caused by Fusarium oxysporum f. sp. fragariae (Fof) severely constrains strawberry production, yet the underlying resistance mechanisms remain unclear.

**Methods:**

A total of 64 strawberry germplasm accessions were evaluated for Fusarium wilt resistance. Integrated physiological and transcriptomic analyses were subsequently performed using the highly resistant cultivar ‘Akihime’ (ZJ) and the highly susceptible cultivar ‘Ning Yu’ (NY).

**Results:**

Resistant resources were abundant, particularly among wild strawberry accessions. Compared with NY, ZJ exhibited higher soluble sugar accumulation, reduced oxidative damage, and increased peroxidase (POD) and phenylalanine ammonia-lyase (PAL) activities. Transcriptomic analyses revealed distinct temporal response patterns: ZJ underwent rapid and extensive transcriptional reprogramming at 24 h post-inoculation, whereas NY showed limited early responses but pronounced changes at 120 h. Gene Ontology (GO) and Kyoto Encyclopedia of Genes and Genomes (KEGG) enrichment analyses indicated that the early response of ZJ was mainly associated with stress-related processes, jasmonic acid-mediated signaling, transmembrane transport, plant–pathogen interaction, mitogen-activated protein kinase (MAPK) signaling, glutathione metabolism, plant hormone signal transduction, and secondary metabolism. Quantitative real-time polymerase chain reaction (qRT-PCR) validation supported the RNA-seq results and identified candidate genes associated with pathogen recognition, signaling, redox regulation, and protein homeostasis.

**Discussion:**

These results indicate that rapid early immune activation and coordinated physiological and metabolic reprogramming are closely associated with strawberry resistance to Fof and provide useful germplasm and candidate genes for future functional validation and resistance breeding.

## 1 Introduction

Strawberry (*Fragaria* spp.) is one of the most economically important horticultural crops worldwide, prized for its excellent nutritional value, high antioxidant content, and appealing sensory characteristics (Fan et al., 2021; Liu et al., 2025). In addition to its commercial value, strawberries play a significant role in the agricultural economies of many countries (Ngouana et al., 2023). In China, strawberry cultivation has grown rapidly, with nearly 200,000 hectares of land dedicated to strawberry farming, making it the largest producer globally (Wei et al., 2024). Among the key regions for strawberry production, Yunnan Province stands out as a strategic hub for summer strawberry cultivation. Leveraging its high-altitude climate, which provides naturally cooler temperatures during the hot summer months, Yunnan has successfully turned this climatic disadvantage into a market advantage. By producing strawberries in the summer off-season, the province captures a unique market window, with nearly 80% of China’s total summer strawberry production taking place in this region (Wan et al., 2021). This strategic advantage has allowed Yunnan to dominate the domestic market for summer strawberries.

However, the long-term sustainability of strawberry farming in China, particularly in high-yield regions like Yunnan, is increasingly threatened by soil-borne diseases, which are exacerbated by intensive monoculture practices and the limited rotation of crops (Zhang et al., 2025). Among these diseases, Fusarium wilt, caused by the soil-borne fungus *Fusarium oxysporum*, has emerged as one of the most devastating pathogens (Yang et al., 2025; Vachev et al., 2025a). Fusarium wilt affects the vascular system of the strawberry plant, causing blockages in the xylem tissues, which disrupts the plant’s ability to transport water and nutrients (Vachev et al., 2025b). As the disease progresses, it leads to wilting, chlorosis, and ultimately plant death (Koike and Gordon, 2015). Given its persistence in the soil, *F. oxysporum* remains a major threat to strawberry production, especially in areas with high plant density and continuous strawberry cultivation. In some fields, disease incidence can exceed 80%, leading to significant yield losses and reduced production quality (Liu et al., 2026).

The challenges posed by Fusarium wilt are compounded by the limited effectiveness of chemical control methods, which are not only environmentally harmful and associated with high residue levels, but also fail to provide long-term protection because the pathogen can persist in the soil for extended periods (Tomar et al., 2026). Biological control has emerged as another sustainable management strategy and has shown potential in suppressing *F. oxysporum* through antagonistic microorganisms and microbiome-mediated disease suppression (Tomar et al., 2026; Zhang et al., 2026). However, its field performance is often inconsistent due to environmental variability and the complexity of soil microbial communities. As a result, there is an increasing demand for durable and sustainable alternatives to manage Fusarium wilt. Genetic resistance to *F. oxysporum* therefore represents one of the most promising solutions (Vachev et al., 2025b). However, developing Fusarium-resistant cultivars requires a comprehensive understanding of the molecular mechanisms underlying disease resistance in strawberry. Despite significant advances in plant pathology and breeding, the genetic and molecular basis of Fusarium resistance in strawberry remains poorly understood, largely due to the complexity of its genome and the difficulty of identifying resistance traits in polyploid crops (Vachev et al., 2025a, b).

Recent advancements in high-throughput transcriptomic technologies have provided valuable tools for studying plant-pathogen interactions at the molecular level (Lyu et al., 2026; Li et al., 2025; Hussain et al., 2026). By comparing the gene expression profiles of resistant and susceptible strawberry cultivars, researchers can identify key genes and biological pathways involved in the plant’s immune response. These pathways often include those related to the production of reactive oxygen species (ROS), phenylpropanoid metabolism, and various plant hormone signaling networks, all of which play critical roles in the plant’s defense against pathogens. Furthermore, understanding how these pathways are activated during early infection stages can offer insights into the mechanisms that allow some strawberry cultivars to resist Fusarium wilt, while others succumb to the disease.

This study aims to perform a comprehensive comparative physiological and transcriptomic analysis of two contrasting cultivars selected from resistance evaluation to *F. oxysporum*. By analyzing gene expression at multiple stages of infection, we aim to uncover the molecular mechanisms that underlie Fusarium resistance in strawberry plants. Specifically, we will focus on identifying differentially expressed genes (DEGs) and key defense-related pathways that are upregulated in resistant cultivars during pathogen attack. The insights gained from this study will contribute to a deeper understanding of strawberry’s defense mechanisms and provide valuable information for breeding programs focused on enhancing Fusarium resistance in strawberry crops.

## 2 Materials and methods

### 2.1 Pathogen cultivation

Strain ZYS0824, a pathogenic *Fusarium oxysporum* isolate, was previously purified from field-collected strawberry plants exhibiting typical Fusarium wilt symptoms, and its pathogenicity was verified via Koch’s postulates.

Genomic DNA extraction, PCR amplification of three loci (ITS, TEF1, Fofra), gel purification and bidirectional Sanger sequencing were all performed by Tsingke Biotechnology Co., Ltd. (Beijing, China). Three primer pairs were used: ITS1 (5′-TCCGTAGGTGAACCTGCGG-3′)/ITS4 (5′-TCCTCCGCTTATTGATATGC-3′), EF1 (5′-ATGGGTAAGGARGACAAGAC-3′)/EF2 (5′-GGARGTACCAGTSATCATGTT-3′), and Fofra-F (5′-CAGACTGGGGTGCTTAAAGTT-3′)/Fofra-R (5′-AACCGCTAGGGTCGTAACAAA-3′) (Suga et al., 2013). Purified PCR products were sequenced on a 3730xl DNA Analyzer (Applied Biosystems, USA). Raw sequencing reads were assembled and trimmed, and the 239 bp Fofra fragment showed 100% sequence identity with the reference sequence of *F. oxysporum* f. sp. *fragariae* (*Fof*). The ITS, TEF1 and Fofra sequences were deposited in GenBank under accession numbers PZ710480, PZ760899 and PZ760900, respectively.

The isolate was cultured on PDA at 28 °C for 5 days, then transferred to PDB and incubated on a ZQTY-70N incubator shaker (Zhichu Instrument Co., Ltd., China) at 180 rpm and 28 °C for another 5 days. The spore suspension was filtered through two layers of gauze. Spore morphology and concentration were detected with a hemocytometer under an Olympus CX43 biological microscope (Olympus, Japan), and adjusted to 2 × 10^7^ spores/mL for inoculation. This culture method was modified from Lei et al. (2019).

### 2.2 Strawberry germplasm and disease resistance evaluation

A total of 64 strawberry germplasm resources, including 41 wild strawberry species and 23 cultivated varieties, were used to assess Fusarium wilt resistance. Healthy, uniform *in-vitro* propagated strawberry seedlings were selected and divided into three replicates, each containing five plants. The plants were randomly assigned to the inoculation and control treatments. The seedlings were inoculated using a root-wounding and root-drenching method. Briefly, approximately one-third of the root system of each seedling was removed using sterile scissors to create wounds, after which the seedlings were transplanted into nursery pots. Each inoculated seedling was then drenched with 100 mL of the *Fof* spore suspension at a concentration of 2 × 10^7^ spores mL^-1^. Control seedlings were subjected to the same root-wounding and transplanting procedures and were drenched with 100 mL of sterile water. The inoculated seedlings were incubated in a growth chamber (Yanghui Instrument, RDN-500B, Ningbo, China) under a 12-hour light/dark cycle, with temperatures set at 25 °C during the night and 30 °C during the day, a light intensity of 20,000 lux, and a relative humidity of 70%. They were watered with sterile water every five days.

**Figure 1:**
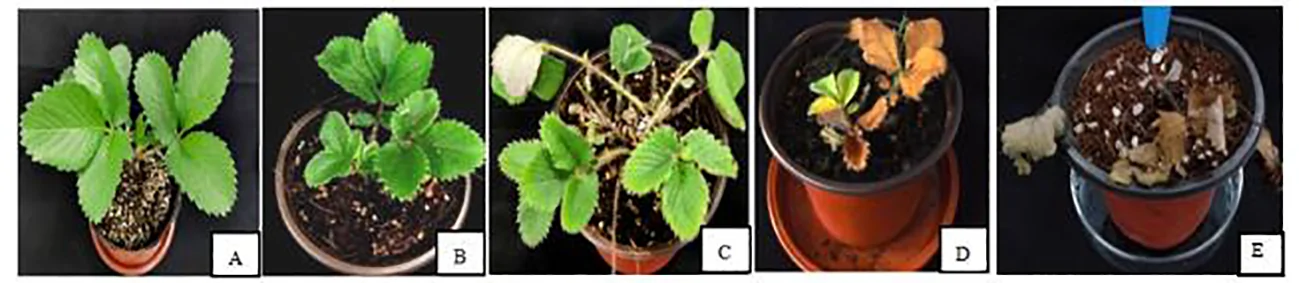
Disease index caused by *Fof* in strawberries. [**(A–E)** represent disease severity grades 0, 1, 2, 3, and 4, respectively].

Two weeks after inoculation, disease incidence and disease index (DI) were recorded, and evaluation was conducted according to the disease severity classification standard outlined in **Figure 1**. The DI was calculated using the following formula: DI = (∑ disease grade × number of plants at each grade) × 100/(total number of plants surveyed × maximum disease grade) (Zhang et al., 2024, 2026). The resistance of the germplasm population was classified based on the disease index at the seedling stage as follows: high resistance (0 < DI ≤ 15), resistance (15 < DI ≤ 35), moderate resistance (35 < DI ≤ 55), susceptibility (55 < DI ≤ 75), and high susceptibility (75 < DI ≤ 100).

### 2.3 Physiological parameters measurement

Physiological analyses were conducted on two octoploid strawberry cultivars-’Akihime’ (ZJ, high resistance) and ‘Ning Yu’ (NY, high susceptibility)-which were sampled at six time points: 0h, 8h, 24h, 72h, 120h, and 168h post-inoculation (hpi). Roots were carefully washed, dried with absorbent paper, and then mixed before being stored at -80 °C for further analysis. Each sample was analyzed in biological triplicates. Physiological indicators such as soluble sugar content, malondialdehyde (MDA) levels, and the activities of defense-related enzymes, including peroxidase (POD) and phenylalanine ammonia-lyase (PAL), were assessed using commercially available kits (Beijing Solarbio Science & Technology Co.,Ltd., China) following the manufacturer’s instructions (Li et al., 2019).

#### 2.3.1 Soluble sugar content measurement

To measure soluble sugar content, 0.1g of strawberry root tissue was homogenized in 1.5 mL of 80% ethanol and incubated at 50 °C for 20 minutes. After cooling, the mixture was centrifuged at 12,000 rpm for 10 minutes, and the supernatant was used for analysis. The absorbance at 620 nm was measured using a spectrophotometer (UV-1800, Shimadzu), and the soluble sugar content was calculated based on the standard curve.

#### 2.3.2 MDA content measurement

MDA content, an indicator of oxidative stress, was determined using the thiobarbituric acid reactive substances (TBARS) assay. Approximately 0.1g of root tissue was ground with 1 mL of extraction buffer, and the homogenate was centrifuged. The supernatant was mixed with thiobarbituric acid and heated at 95 °C for 30 minutes. The absorbance was measured at 532 nm, and the MDA content was calculated using the formula provided in the kit’s protocol.

#### 2.3.3 POD and PAL activity measurement

For POD and PAL activity assays, 0.1g of root tissue was homogenized in 1 mL of cold extraction buffer. The resulting homogenate was centrifuged, and the supernatant was used to assess enzyme activities. POD activity was measured by monitoring the increase in absorbance at 470 nm, which corresponds to the formation of a reddish-brown compound in the reaction. For PAL activity, the change in absorbance at 290 nm was recorded as the enzyme catalyzed the conversion of phenylalanine to cinnamic acid.

### 2.4 RNA extraction and RNA sequencing

Transcriptomic analysis was performed on the root tissues of the high-resistant cultivar ZJ and the high-susceptible cultivar NY at three different infection time points (0h, 24h, and 120h), with each sample containing three biological replicates, and physiological indicators were sampled simultaneously. Samples were sent to Metware Biotechnology Co., Ltd. (Wuhan, China) for transcriptome sequencing.

RNA extraction was performed using a plant total RNA extraction kit on strawberry root tissues. The RNA concentration was accurately measured using a Qubit 4.0 Fluorometer, while RNA integrity was assessed using the Qsep400 Bioanalyzer. Only RNA samples that passed quality control were used for further analysis.

For library preparation, mRNA was enriched from the total RNA using poly-T oligo-dT magnetic beads, and first-strand cDNA was synthesized via reverse transcription. The cDNA was then purified and subjected to end repair to prepare for sequencing library construction. After completing the library construction, the quality of the library was assessed, and only those meeting the required standards were used for sequencing.

Sequencing was conducted on the Illumina 6000 platform, which generated high-quality transcriptome data. The raw data were first processed using fastp to remove low-quality reads and adapters, yielding high-quality clean data for further analysis (Chen et al., 2018). Subsequently, the clean reads were aligned to the strawberry reference genome (*Fragaria* × *ananassa* Yanli Genome v1.0), available at https://www.rosaceae.org/Analysis/14723107, using HISAT2 for sequence alignment (Muley, 2025). Gene expression was quantified at the count level using featureCounts, providing an accurate measurement of gene expression based on the aligned reads (Liao et al., 2014).

### 2.5 Differential expression analysis and functional annotation

Differential expression analysis of genes was performed using the DESeq2 package in R. Genes with |log2(fold change)| ≥ 1 and q-value ≤ 0.05 were considered as differentially expressed genes(DEGs). To characterize biological functions and metabolic pathways enriched among DEGs, Gene Ontology (GO) and Kyoto Encyclopedia of Genes and Genomes (KEGG) enrichment analyses were performed. All enrichment analyses were run in R v4.2.2 using clusterProfiler v4.6.0. Specifically, GO enrichment was implemented with the enrichGO function, and KEGG enrichment was achieved via the enrichKEGG function. Enrichment significance was assessed using hypergeometric tests at the GO term and KEGG pathway levels, respectively. Local offline GO and KEGG databases were employed to guarantee stable and reproducible enrichment outcomes, and all annotated genes from the strawberry reference genome were used as the universal background gene set for enrichment.

Separate multiple testing correction approaches were applied: Bonferroni correction was performed for KEGG enrichment results, whereas Benjamini–Hochberg (BH) correction was used for GO enrichment P-values. GO terms and KEGG pathways with a *p*-value ≤ 0.05 were considered significantly enriched.

### 2.6 qRT-PCR validation

RNA was extracted as described previously. cDNA was synthesized using the PrimeScript One Step RT-PCR Kit Ver.2 (Dye Plus). qPCR primers were designed using the NCBI Primer-BLAST tool, ensuring primer specificity and optimal melting temperature. Gene expression levels were calculated using the 2^(-ΔΔCT) method (Livak and Schmittgen, 2001).

### 2.7 Statistical analysis

All experiments were conducted in three biological replicates, and statistical significance was determined using one-way analysis of variance (ANOVA), followed by Tukey’s test to compare group means. Statistical analyses were performed using SPSS version 25.0 (IBM, USA). A p-value of less than 0.05 was considered statistically significant.

## 3 Results and discussion

### 3.1 Resistance evaluation of strawberry germplasm

A total of 64 strawberry germplasm resources, comprising 41 wild strawberry species and 23 cultivated varieties, were evaluated for resistance to *Fof* (**Table 1**). After inoculation, the germplasm resources exhibited marked variation in resistance levels: 39 materials displayed high resistance, 3 were classified as resistant, 8 showed moderate resistance, 5 were susceptible, and 9 exhibited high susceptibility. Overall, highly resistant materials were predominant, particularly among wild strawberry species. This enrichment of resistance in wild germplasm suggests the presence of abundant and valuable genetic resources for disease resistance. Notably, during the evolutionary history of octoploid strawberry, extensive gene loss has occurred, which is considered one of the major driving forces underlying subgenome dominance and biased gene expression (Jin et al., 2023). Such evolutionary processes may have contributed to the diversification and retention of resistance-related genes in specific subgenomes, thereby shaping the observed resistance variation among strawberry germplasm. Collectively, these findings indicate that the evaluated germplasm pool provides a solid genetic foundation for future strawberry resistance breeding and for further investigation into the molecular mechanisms underlying disease resistance. In particular, the highly resistant wild accessions identified in this study may serve as valuable donor materials for broadening the genetic basis of Fusarium wilt resistance in cultivated strawberry.

**Table 1:** Identification of resistance to strawberry Fusarium wilt in tested strawberry germplasm.

Ploidy	Species (Latin name)	Accession ID	Disease incidence	Disease index	Resistance category	Ploidy	Species (Latin name)	Accession ID	Disease incidence	Disease index	Resistance category
Diploid (2x)	*F. vesca L.*	1	40%	10	High resistance	Octoploid (8x)	*F. chiloensis (L.) Duch.*	45	0%	0	High resistance
		2	60%	45	Moderate resistance			46-1	100%	100	High susceptibility
		3	0%	0	High resistance			46-2	100%	100	High susceptibility
	*F. nilgerrensis* Schlect.	5	0%	0	High resistance		*F. virginiana Duch.*	47	100%	93.3	High susceptibility
		6	0%	0	High resistance			48	0%	100	High resistance
		7	0%	0	High resistance			49	100%	71.1	Susceptibility
	*F. viridis Duch.*	8	40%	20	Resistance			50	100%	64.4	Susceptibility
		9	60%	55	Moderate resistance		*F. × ananassa Duch.*	Albion	0%	0	High resistance
		10	33%	11.7	High resistance			Camarosa	80%	65	Susceptibility
	*F. nubicola Lindl.*	13	0%	0	High resistance			Ssanta	100%	45	Moderate resistance
		14	0%	0	High resistance			Angel Eight	100%	70	Susceptibility
	*F. pentaphylla Lozinsk.*	16	0%	0	High resistance			Feiyu	80%	40	Moderate resistance
		17	0%	0	High resistance			Benihoppe	100%	45	Moderate resistance
		18	60%	25	Resistance			Lvfeng	40%	15	High resistance
		19	40%	13.3	High resistance			Snow Princess	100%	84.4	High susceptibility
		20	0%	0	High resistance			Yukiusagi	100%	77.8	Moderate resistance
		21	0%	0	High resistance			Seolhyang	60%	38.3	High susceptibility
		22	0%	0	High resistance			Kaorino	100%	87.5	High susceptibility
		23	0%	0	High resistance			Yutu	100%	100	High susceptibility
	*F. mandschurica Staudt*	24	0%	0	High resistance			Yue Xiu	0%	0	High resistance
		25	0%	0	High resistance			Akihime	0%	0	High resistance
	*F. nipponica Lindl.*	27	0%	0	High resistance			Sweet Charlie	0%	0	High resistance
	*F. yezoensis Hara.*	29	0%	0	High resistance			San Andreas	80%	45	Moderate resistance
Tetraploid (4x)	*F. orientalis Lozinsk.*	31	0%	0	High resistance			Qiuyi	33%	13.3	High resistance
	*F. moupinensis (Franch) Card.*	32	0%	0	High resistance			Qiuxiang	0%	0	High resistance
		33	0%	0	High resistance			Qiuwen	0%	0	High resistance
		34	0%	0	High resistance			Qiuhong	67%	36.7	Moderate resistance
	*F. corymbosa Lozinsk.*	36	0%	0	High resistance			Ning Yu	100%	100	High susceptibility
		37	67%	25	Resistance			Miaoxiang	110%	90	High susceptibility
	*F. gracilis Lozinsk.*	39	0%	0	High resistance			Monterey	100%	63.3	Susceptibility
	*F. tibetica Staudt et Dickoré*	40	33%	13.3	High resistance	Decaploid (10x)	*F. iturupensis Staudt*	52	0%	0	High resistance
		41	0%	0	High resistance						
		42	20%	8.3	High resistance						

For practical application, the high-resistant cultivar ZJ, commonly known as “milk strawberry,” is highly favored by consumers and has become a popular cultivar in production (Wang et al., 2024). In contrast, the highly susceptible cultivar NY is a new early-maturing, forcing-cultivated variety derived from a cross between ZJ (as the paternal parent) and ‘Xingxiang’ (as the maternal parent). NY exhibits excellent fruit quality but has weaker disease resistance. The significant difference in disease resistance between these two cultivars makes them ideal for studying the response mechanisms of strawberry to *Fof*. By comparing these two genetically related but distinctly resistant cultivars, physiological and molecular responses during *Fof* infection can be examined, helping to reveal the mechanisms underlying strawberry disease resistance and providing a theoretical basis for disease-resistant breeding.

### 3.2 Physiological and biochemical differences in high-resistance and high-susceptibility strawberries

**Figure 2:**
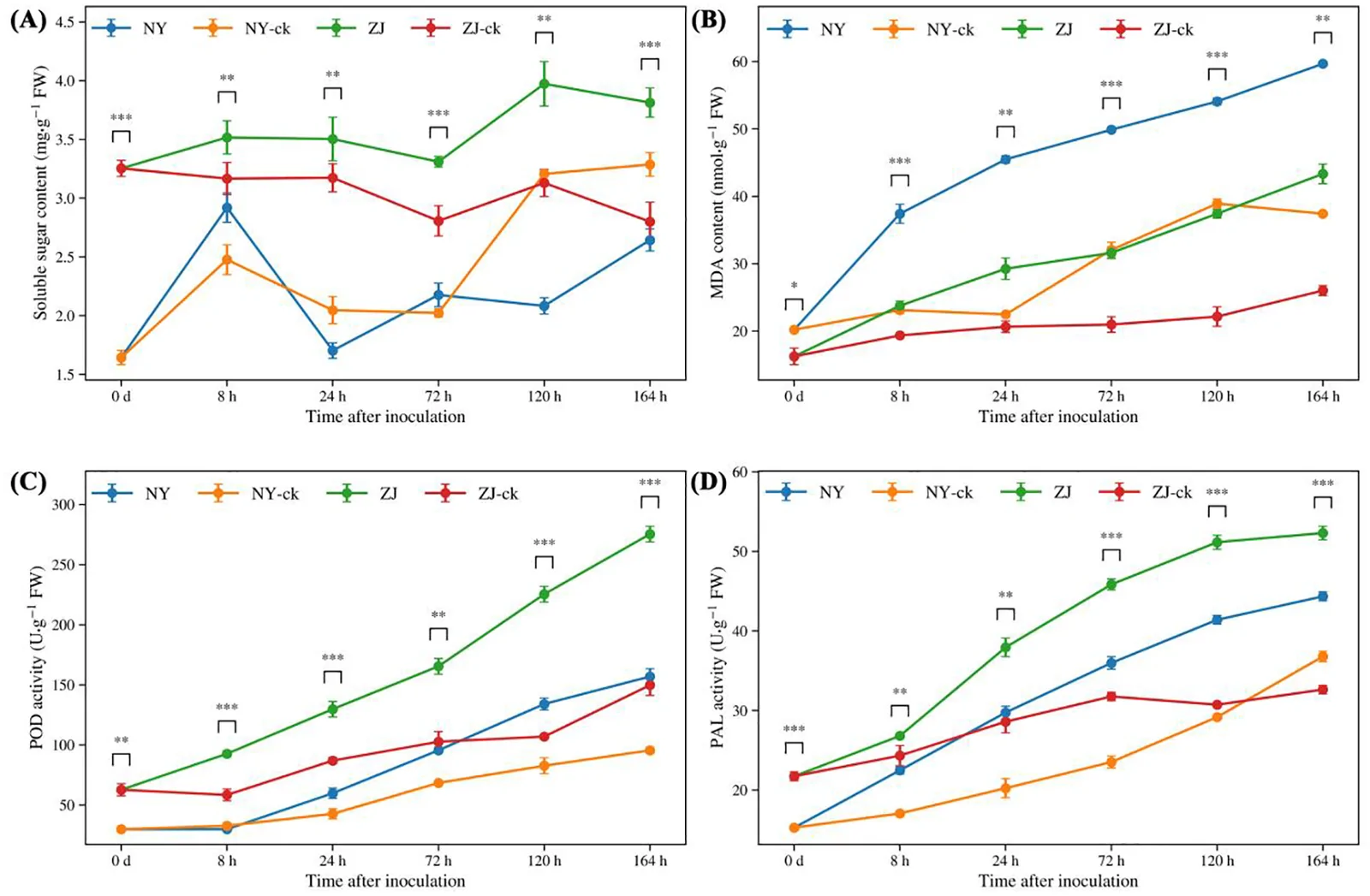
Physiological and biochemical differences between NY and ZJ following *Fof* infection, including soluble sugar content **(A)**, malondialdehyde (MDA) content **(B)**, peroxidase (POD) activity **(C)**, and phenylalanine ammonia-lyase (PAL) activity **(D)**. ZJ, the highly resistant cultivar ‘Akihime’; NY, the highly susceptible cultivar ‘Ning Yu’. Data are presented as mean ± SD of three biological replicates. Asterisks indicate significant differences between ZJ and NY at the same time point based on Welch’s t-test (*p < 0.05, **p < 0.01, ***p < 0.001).

After inoculation with *Fof*, significant differences in physiological indicators were observed between the high-resistant cultivar ZJ and the susceptible cultivar NY. Soluble sugars, which function as osmoregulatory agents and signaling molecules, play a key role in plants’ response to environmental stress and adaptation (Wang et al., 2018; Chen et al., 2024). The high resistance of ZJ is closely associated with its soluble sugar accumulation pattern (**Figure 2A**). Following infection, the soluble sugar content in ZJ steadily increased, reaching its peak in the later stages (120 hours). This timely and sustained sugar supply supports enhanced secondary metabolic activity and meets the energy demands required to alleviate oxidative stress and cellular damage. In comparison, ZJ consistently exhibited higher soluble sugar levels than NY, suggesting that ZJ may rely on a stronger secondary metabolism and energy supply to cope with pathogen-induced cellular damage, highlighting the role of soluble sugars in boosting disease resistance mechanisms.

As a product of membrane lipid peroxidation, malondialdehyde (MDA) reflects the extent of oxidative damage and can be used as an indirect indicator of cellular stress severity (Zhong et al., 2023; Song et al., 2025). After infection, both ZJ and NY showed elevated oxidative stress, as evidenced by a time-dependent accumulation of MDA (**Figure 2B**). However, NY exhibited consistently higher MDA levels than ZJ, particularly at 72 h and 120 h post-inoculation, indicating more pronounced lipid peroxidation and membrane injury in NY at later stages of infection. In contrast, the lower (or more slowly increasing) MDA content in ZJ suggests that this resistant cultivar more effectively limits pathogen-induced oxidative damage, likely by maintaining membrane integrity and/or activating stronger antioxidant and detoxification systems. This reduced lipid peroxidation may contribute to ZJ’s higher resistance to *Fof*.

POD is an important defense enzyme that catalyzes the final step of lignin formation and converts phenolic compounds into highly toxic quinone derivatives (Moosa et al., 2022). PAL plays a key role in regulating the phenolic metabolic pathway and catalyzes the conversion of phenylalanine to trans-cinnamic acid (Moosa et al., 2022). Additionally, PAL is a precursor for scoparone and scopoletin, both of which have been reported to possess antifungal properties against various fungal pathogens (Droby et al., 2002). In terms of POD activity, ZJ showed a rapid increase after 24 hours and maintained a high level, demonstrating a significantly stronger ability to scavenge reactive oxygen species (ROS) compared to NY (**Figure 2C**). As for PAL levels, ZJ exhibited significantly higher PAL activity at all time points post-infection, particularly reaching notable peaks at 72 and 120 hours (**Figure 2D**). This suggests that ZJ mounts a stronger induced defense response against *Fof* than NY. This response is consistent with the reported roles of POD in reactive oxygen species detoxification and lignin formation and of PAL in phenylpropanoid-derived defense metabolism (Moosa et al., 2022; Droby et al., 2002). Therefore, the higher POD and PAL activities in ZJ may contribute to resistance by limiting oxidative damage and strengthening structural and chemical defense barriers.

In summary, significant physiological and biochemical differences were observed between the high-resistant cultivar ZJ and the susceptible cultivar NY after inoculation with *Fof*. ZJ exhibited higher accumulation of soluble sugars, supporting enhanced secondary metabolism and energy supply to mitigate oxidative stress and cellular damage. In contrast, NY showed higher MDA levels, especially at the later stages of infection, indicating more severe lipid peroxidation and membrane damage. ZJ also showed significantly higher POD and PAL activities, particularly at 72 and 120 hours post-infection. These results suggest that ZJ mounts a stronger defense response through rapid reaction and sustained regulatory mechanisms, contributing to its superior resistance to the pathogen. Overall, the differential accumulation of soluble sugars, MDA, POD, and PAL highlights the biochemical basis for the resistance differences between ZJ and NY, offering valuable insights into the mechanisms underlying strawberry disease resistance.

### 3.3 Transcriptome data collection and quality control

For transcriptome analysis, root samples were collected at three representative time points (0h, 24h, and 120h) to capture distinct stages of the strawberry response to *Fof*. The 0h time point served as the baseline prior to pathogen exposure, enabling characterization of cultivar-specific gene expression under non-stressed conditions. The 24h time point represented the early infection stage, allowing detection of rapid defense activation and associated signaling dynamics, whereas 120h reflected a later stage when more stable regulatory and adaptive responses are established. This design facilitates a comprehensive assessment of transcriptional reprogramming from early recognition to longer-term defense regulation.

To investigate transcriptomic variation between the high-resistant cultivar ZJ and the susceptible cultivar NY, RNA-seq was performed at 0h, 24h, and 120h with three biological replicates per treatment, yielding 30 libraries and 1.593 billion raw reads. After removal of adapter sequences and low-quality reads, each library retained 44.38-59.48 million clean reads, totaling 94 GB of clean bases. Overall sequencing quality was high, with an error rate consistently below 0.01%; Q20 and Q30 values ranged from 97.32% to 97.85% and from 91.90% to 93.33%, respectively (**Table 2**). In addition, GC content varied from 47.00% to 53.00% (**Table 2**), indicating no obvious base-composition bias. Collectively, these metrics demonstrate that the RNA-seq datasets are of sufficient quality to support downstream analyses and candidate gene discovery.

**Table 2:** Samples sequencing data assessment and assembly statistics table.

Sample	Raw reads	Clean reads	Clean base (G)	Error rate (%)	Q20 (%)	Q30 (%)	GC content (%)
ZJ-0d-1	60581282	59477954	8.92	0.01	97.58	92.65	50.53
ZJ-0d-2	54861592	54095664	8.11	0.01	97.77	93.17	51.08
ZJ-0d-3	52009132	51193248	7.68	0.01	97.70	92.98	50.58
ZJ-CK-1d-1	60518592	59644546	8.95	0.01	97.72	92.99	50.94
ZJ-CK-1d-2	54268162	53536094	8.03	0.01	97.38	92.10	53.00
ZJ-CK-1d-3	54503020	53815170	8.07	0.01	97.46	92.31	52.62
ZJ-1d-1	57556130	56738586	8.51	0.01	97.74	93.06	50.70
ZJ-1d-2	57102350	56310958	8.45	0.01	97.64	92.73	50.22
ZJ-1d-3	49274562	48599834	7.29	0.01	97.61	92.62	51.67
ZJ-5d-1	48872934	48197990	7.23	0.01	97.47	92.31	50.88
ZJ-5d-2	46117950	45430226	6.81	0.01	97.32	91.90	50.19
ZJ-5d-3	50812466	50097994	7.51	0.01	97.72	92.99	50.78
ZJ-CK-5d-1	51619574	50866204	7.63	0.01	97.71	92.95	50.19
ZJ-CK-5d-2	54493120	53761532	8.06	0.01	97.63	92.76	50.38
ZJ-CK-5d-3	50268156	49584070	7.44	0.01	97.58	92.59	50.50
NY-0d-1	55050058	54126776	8.12	0.01	97.67	92.87	50.85
NY-0d-2	58372082	57562852	8.63	0.01	97.69	92.95	50.70
NY-0d-3	56796348	56057780	8.41	0.01	97.85	93.33	50.47
NY-CK-1d-1	44963406	44377316	6.66	0.01	97.64	92.94	47.87
NY-CK-1d-2	48102646	47458636	7.12	0.01	97.37	92.09	52.65
NY-CK-1d-3	48317188	47738734	7.16	0.01	97.45	92.26	51.4
NY-1d-1	51456632	50747254	7.61	0.01	97.61	92.73	50.63
NY-1d-2	50629024	49947564	7.49	0.01	97.58	92.59	50.54
NY-1d-3	53389112	52600690	7.89	0.01	97.72	93.14	47.00
NY-CK-5d-1	54888806	54155972	8.12	0.01	97.65	92.79	49.97
NY-CK-5d-2	54871428	54162606	8.12	0.01	97.72	92.99	49.69
NY-CK-5d-3	53294684	52632890	7.89	0.01	97.83	93.25	49.95
NY-5d-1	53046396	52370896	7.86	0.01	97.47	92.33	50.79
NY-5d-2	54385692	53722206	8.06	0.01	97.81	93.24	50.89
NY-5d-3	52629462	51950898	7.79	0.01	97.73	93.04	50.88

NY, the susceptible cultivar ‘Ning Yu’; ZJ, high-resistant cultivar ‘Akihime’.

### 3.4 DEGs in response to *Fof* infection

**Figure 3:**
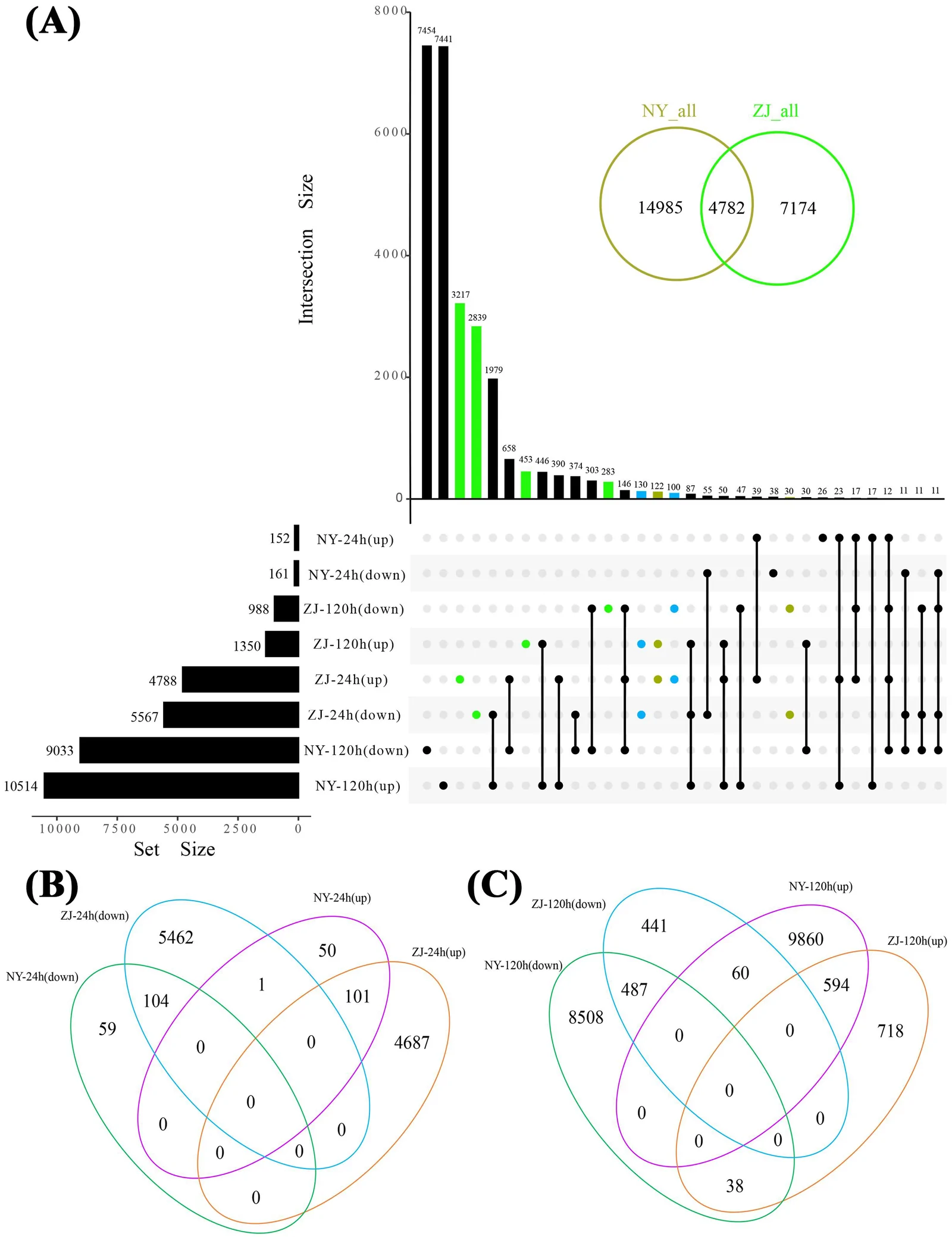
Distribution and overlap of differentially expressed genes (DEGs) in the susceptible cultivar ‘Ning Yu’ (NY) and the resistant cultivar ‘Akihime’ (ZJ) following *Fof* infection. **(A)** UpSet plot showing the intersections among upregulated and downregulated DEG sets at 24 and 120 h post-inoculation relative to the corresponding time-matched controls. The vertical bars represent the intersection size (number of DEGs), the horizontal bars represent the set size (number of DEGs), and the connected dots indicate the DEG sets included in each intersection. **(B, C)** Venn diagrams showing cultivar-specific and shared upregulated and downregulated DEGs at 24 h **(B)** and 120 h **(C)**, respectively.

To elucidate the mechanisms underlying strawberry resistance to *Fof*, we compared the DEGs in the resistant cultivar ZJ and the susceptible cultivar NY at 24 h and 120 h post-inoculation. The two cultivars exhibited markedly different transcriptional response magnitudes and temporal patterns. In NY, only 152 genes were upregulated and 163 genes were downregulated at 24 h, whereas transcriptional reprogramming became much more pronounced at 120 h, with 10,514 genes upregulated and 9,033 genes downregulated. In contrast, ZJ displayed extensive transcriptional changes as early as 24 h, with 4,788 upregulated and 5,567 downregulated genes; by 120 h, the number of DEGs decreased to 1,350 upregulated and 988 downregulated genes (**Figure 3**). Overall, 11,956 and 19,767 DEGs were identified in ZJ and NY, respectively, indicating substantial differences in the scope and intensity of pathogen-responsive transcription between the two cultivars.

Venn diagram analyses further revealed pronounced cultivar-specific transcriptional responses at both infection stages. At 24 h, NY contained only 50 uniquely upregulated and 59 uniquely downregulated genes, whereas ZJ showed 4,687 uniquely upregulated and 5,462 uniquely downregulated genes (**Figure 3A**). At 120 h, the number of cultivar-specific DEGs increased dramatically in NY, reaching 9,860 uniquely upregulated and 8,508 uniquely downregulated genes, while ZJ exhibited comparatively fewer unique DEGs (718 upregulated and 441 downregulated; **Figure 3B**). Collectively, these results indicate a clear temporal divergence in defense activation: ZJ rapidly initiates large-scale transcriptional reprogramming during the early infection stage (24 h), which likely facilitates earlier activation of defense-related pathways and establishment of an effective resistance state, consistent with the physiological and biochemical evidence of faster and stronger defense responses. In contrast, NY displays a delayed and more extensive transcriptional response at the later stage (120 h), which may reflect a secondary, stress- or damage-associated response after substantial pathogen progression, thereby contributing to its overall susceptibility compared with ZJ.

### 3.5 GO enrichment analysis of DEGs

**Figure 4:**
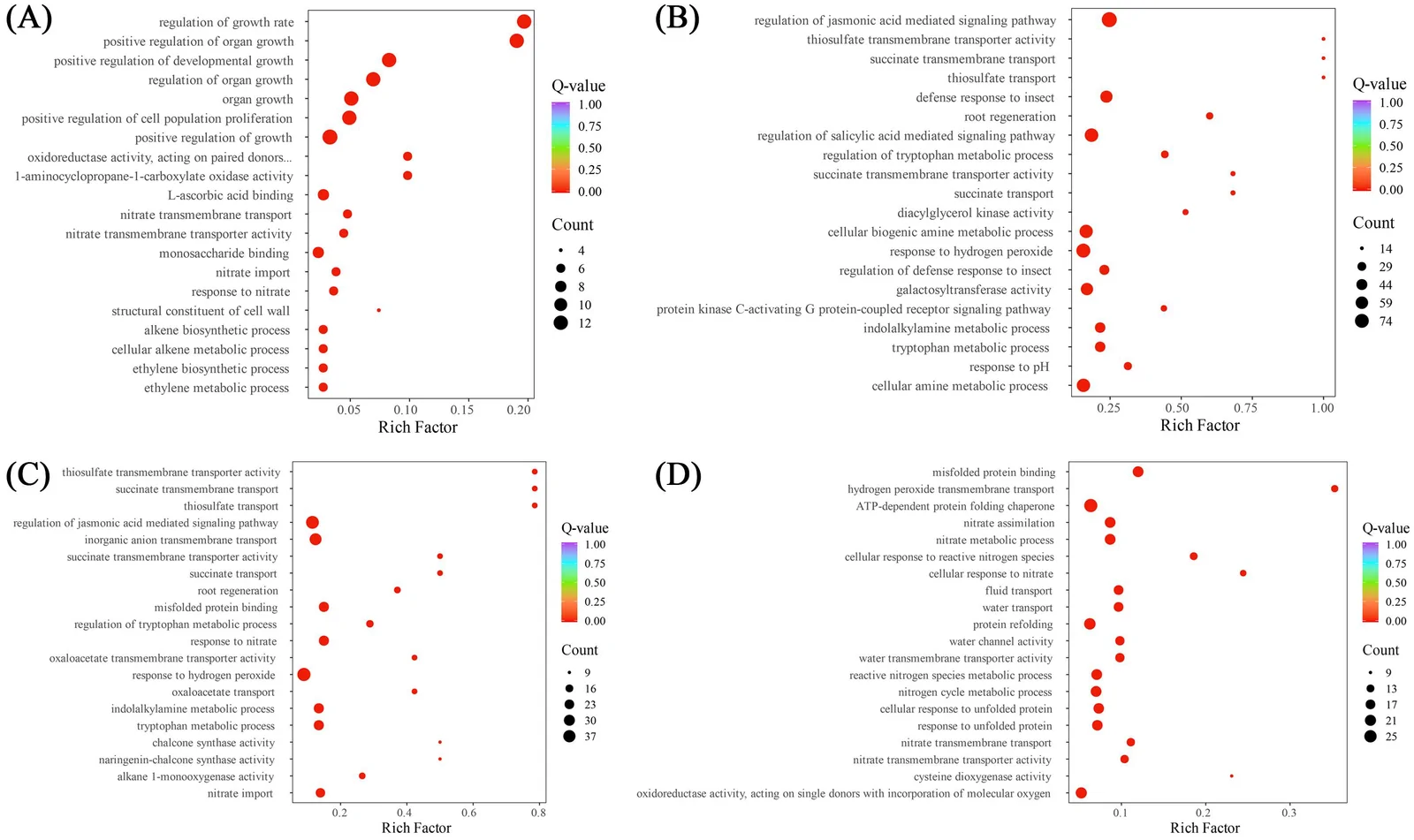
Gene Ontology (GO) enrichment scatter plots of differentially expressed genes (DEGs) in NY and ZJ before and after *Fof* infection. **(A)** NY, 24 h post-inoculation vs. 24 h control; **(B)** NY, 120 h post-inoculation vs. 120 h control; **(C)** ZJ, 24 h post-inoculation vs. 24 h control; **(D)** ZJ, 120 h post-inoculation vs. 120 h control. ZJ, the highly resistant cultivar ‘Akihime’; NY, the highly susceptible cultivar ‘Ning Yu’.

To elucidate the molecular mechanisms underlying the contrasting responses of resistant and susceptible cultivars to *Fof* infection, GO and KEGG enrichment analyses were conducted on cultivar-specific DEGs identified in ZJ and NY at 24 h and 120 h post-inoculation. GO enrichment analysis was categorized into three domains: Biological Process (BP), Cellular Component (CC), and Molecular Function (MF) (Wu et al., 2025; Wang et al., 2023). The top 20 significantly enriched GO terms were visualized using bubble plots (**Figure 4**).

At the early stage of infection (24 h), ZJ exhibited significant enrichment in multiple stress- and transport-related biological processes (**Figure 4C**), including regulation of jasmonic acid-mediated signaling pathway (GO:2000022), succinate transmembrane transport (GO:0071422), thiosulfate transport (GO:0015709), inorganic anion transmembrane transport (GO:0098661), succinate transport (GO:0015744), and root regeneration (GO:0062211). In the MF category, thiosulfate transmembrane transporter activity (GO:0015117) was also significantly enriched. The enrichment of jasmonic acid-mediated signaling in ZJ suggests that hormone-associated immune regulation may be rapidly activated in the resistant cultivar during the early stage of infection. In contrast, the absence of comparable early enrichment in NY indicates that hormone-related defense regulation may be delayed or weaker in the susceptible cultivar. In contrast, NY primarily showed enrichment in growth- and development-related processes (**Figure 4A**), such as regulation of growth rate (GO:0040009), positive regulation of organ growth (GO:0046622), and positive regulation of developmental growth (GO:0048639).

At 120 h post-inoculation, the enriched metabolic pathways in NY resembled those observed in ZJ during the early infection stage (**Figure 4B**), suggesting a delayed activation of defense-related responses in the susceptible cultivar. In contrast, ZJ at this stage predominantly exhibited enrichment in pathways associated with protein quality control and stress adaptation (**Figure 4D**), including misfolded protein binding (GO:0051787), ATP-dependent protein folding chaperone activity (GO:0140662), as well as nitrate assimilation (GO:0042128) and nitrate metabolic process (GO:0042126).

These GO enrichment results should be interpreted as pathway-level evidence for functional divergence between the two cultivars rather than as a complete reconstruction of gene-level expression patterns within each biological process. Nevertheless, the contrasting enrichment patterns provide important clues regarding the timing of defense activation. The enrichment of jasmonic acid-mediated signaling, stress-related processes, and transport-related functions in ZJ at 24 h post-inoculation suggests that the resistant cultivar rapidly redirects cellular regulation from basal growth toward defense-related responses. By contrast, the predominance of growth- and development-related GO terms in NY at the same stage indicates that the susceptible cultivar may not efficiently prioritize defense activation during the early phase of infection. Collectively, these results indicate that the resistant cultivar ZJ initiates a broader range of stress-responsive and defense-related pathways at an earlier stage of infection compared to NY, thereby enabling a more rapid and effective response to *Fof* invasion. The pronounced differences in enriched pathways between the two cultivars across infection stages highlight distinct temporal defense strategies and regulatory priorities.

### 3.6 KEGG pathway enrichment analysis of DEGs

**Figure 5:**
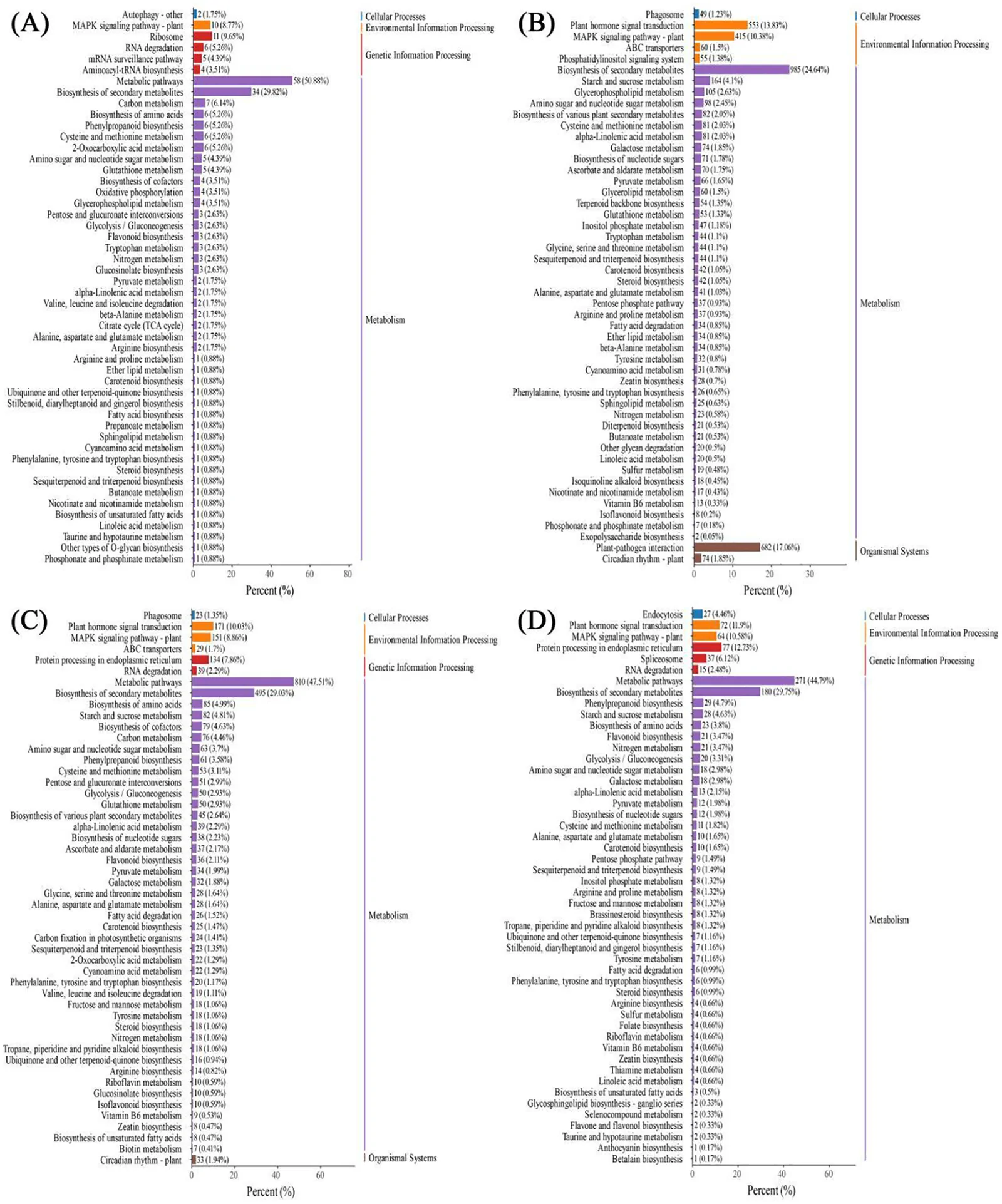
Kyoto Encyclopedia of Genes and Genomes (KEGG) pathway enrichment percent bar graph of differentially expressed genes (DEGs) in NY and ZJ before and after *Fof* infection. **(A)** NY, 24 h post-inoculation vs. 24 h control; **(B)** NY, 120 h post-inoculation vs. 120 h control; **(C)** ZJ, 24 h post-inoculation vs. 24 h control; **(D)** ZJ, 120 h post-inoculation vs. 120 h control. ZJ, the highly resistant cultivar ‘Akihime’; NY, the highly susceptible cultivar ‘Ning Yu’.

At 24 h post-inoculation, NY-specific DEGs were primarily enriched in transporter-related pathways, suggesting that its early response is largely limited to material transport and basic physiological adjustment (**Figure 5A**). In contrast, ZJ exhibited a markedly stronger immunity-related transcriptional reprogramming at this stage. Specifically, ZJ-specific DEGs were significantly enriched in multiple canonical defense- and secondary metabolism-associated pathways, including flavonoid biosynthesis, phenylpropanoid biosynthesis, MAPK signaling pathway-plant, glutathione metabolism, alpha-linolenic acid metabolism, plant hormone signal transduction, plant-pathogen interaction, and sesquiterpenoid and triterpenoid biosynthesis (**Figure 5C**).

Although the present analysis does not provide a complete gene-by-gene expression profile for each enriched pathway, the KEGG enrichment results reveal the major functional categories preferentially activated in the resistant cultivar. The enrichment of plant-pathogen interaction and MAPK signaling pathways suggests early activation of pathogen perception and downstream immune signaling in ZJ. The enrichment of glutathione metabolism is consistent with enhanced redox regulation, while the enrichment of plant hormone signal transduction indicates that hormone-mediated defense regulation may contribute to the early response. In addition, the enrichment of phenylpropanoid, flavonoid, and terpenoid biosynthesis pathways suggests that secondary metabolism may participate in strengthening antimicrobial and stress-protective responses. These enrichment patterns collectively indicate that ZJ rapidly activates interconnected immune signaling, antioxidant regulation, and metabolic defense processes during the early phase of infection.

At 120 h post-inoculation, the enrichment profile of NY-specific DEGs shifted markedly toward pathways associated with lipid metabolism and stress responses, including alpha-linolenic acid metabolism, glycerolipid metabolism, plant–pathogen interaction, and secretion systems. This shift suggests that the late transcriptional response of NY is more closely associated with pathogen progression, membrane remodeling, and stress- or damage-induced processes (**Figure 5B**). In contrast, ZJ-specific DEGs at this stage remained predominantly enriched in secondary metabolism pathways, such as flavonoid, phenylpropanoid, carotenoid, and terpenoid biosynthesis (**Figure 5D**), indicating sustained activation of antimicrobial metabolite production during the later stages of infection. Notably, plant hormone signal transduction was consistently enriched in ZJ, suggesting that hormone-mediated defense regulation may contribute to both early and sustained resistance responses.

Collectively, these pathway-level enrichment results reveal a clear stage-dependent divergence between the two cultivars. ZJ showed early enrichment of immune- and secondary metabolism-related pathways at 24 h post-inoculation, whereas NY showed limited early activation and more pronounced enrichment of stress- and defense-related pathways at 120 h post-inoculation. This temporal pattern, characterized by rapid early pathway activation in ZJ versus delayed pathway activation in NY, suggests that timely pathogen perception and prompt immune activation are critical for effective resistance. Therefore, while detailed expression profiling of all genes within these pathways remains to be further investigated, the current KEGG enrichment results support the conclusion that the timing of pathway activation is closely associated with Fusarium wilt resistance in strawberry.

### 3.7 qRT-PCR validation

**Figure 6:**
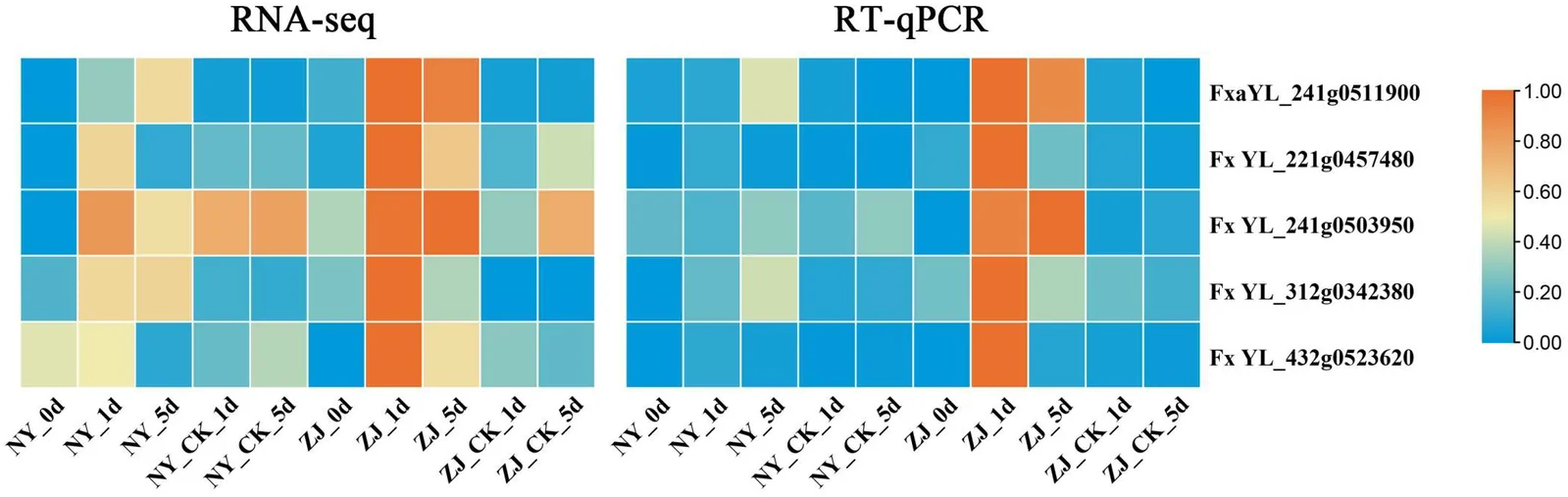
qRT-PCR analysis of differentially expressed genes (DEGs). ZJ, the highly resistant cultivar ‘Akihime’; NY, the highly susceptible cultivar ‘Ning Yu’.

To validate the reliability of the RNA-seq data and further prioritize key genes associated with strawberry resistance to Fusarium wilt, we selected 5 pathogen-responsive genes from the DEGs and performed qRT-PCR to examine their relative expression levels in the highly resistant cultivar ZJ and the highly susceptible cultivar NY across different infection stages (**Figure 6**). Overall, the qRT-PCR results were highly consistent with the expression trends observed in RNA-seq, indicating that the transcriptome sequencing data were robust and reproducible. These candidates displayed distinct expression profiles during the early and/or mid–late stages of infection and are implicated in key processes such as pathogen recognition, signal transduction, antioxidant defense, and maintenance of protein homeostasis, suggesting that they may play central roles in strawberry roots during *Fof* infection.

First, *FxaYL_241g0511900* was rapidly upregulated in ZJ at the early infection stage (24 h), whereas its expression became obvious only at the later stage (120 h) in NY (**Figure 6**). This gene contains a chitin-binding domain, which can recognize and bind chitin, a major component of fungal cell walls. Therefore, it may participate in pathogen-associated molecular pattern (PAMP) perception and immune activation, facilitating rapid initiation of defense responses in the resistant cultivar. Second, *FxaYL_432g0523620* showed high expression in ZJ at the early infection stage but was nearly undetectable in NY throughout the infection period (**Figure 6**). This gene encodes a heat shock protein (HSP), which functions as a molecular chaperone to promote proper protein folding and alleviate stress-induced protein damage, thereby maintaining cellular homeostasis and enhancing tolerance to adverse conditions such as pathogen invasion.

Third, *FxaYL_312g0342380* exhibited significantly higher expression in ZJ than in NY during the early infection stage (**Figure 6**) and encodes a glutathione S-transferase (GST). GSTs are multifunctional enzymes involved not only in detoxification but also in regulating glutathione-dependent antioxidant systems to maintain cellular redox balance, thereby alleviating oxidative stress induced by pathogen infection. This is consistent with the ROS-related defense differences reflected by the physiological indicators described above. Fourth, *FxaYL_221g0457480* was markedly expressed in ZJ at the early stage, whereas its expression remained extremely low in NY across the entire infection period (**Figure 6**). This gene contains an HSF-type DNA-binding domain and is predicted to encode a heat shock transcription factor (HSF). As a TF-related candidate gene, *FxaYL_221g0457480* may contribute to the contrasting responses between ZJ and NY by regulating HSP family genes and other stress-responsive genes involved in protein homeostasis, stress adaptation, and possibly immune signaling. Its strong induction in ZJ but extremely low expression in NY suggests that early activation of TF-mediated stress regulation may be associated with resistance. Finally, *FxaYL_241g0503950* maintained relatively high expression in ZJ at both the early and later stages and was significantly higher than that in NY (**Figure 6**). This gene encodes a receptor-like kinase (RLK), a key plasma membrane receptor that can perceive pathogen-derived signals and initiate intracellular signaling cascades, thereby triggering immune responses and acting as a critical node linking “pathogen perception–signal transduction–defense execution.”

Taken together, these five candidate genes showed earlier, stronger, and/or more sustained induction in the resistant cultivar, and their predicted functions span multiple essential components of plant immunity, including pathogen recognition, defense signaling, antioxidant regulation, and protein homeostasis. From a breeding perspective, these genes represent preliminary candidates for the development of molecular markers and the screening of Fusarium wilt-resistant strawberry materials. However, their direct roles in resistance require further confirmation through functional analyses, such as gene overexpression, silencing, or gene editing. When integrated with the physiological results, these transcriptomic and qRT-PCR findings provide a preliminary link between molecular responses and defense-related physiological changes. The higher expression of genes associated with pathogen recognition and signal transduction in ZJ may contribute to earlier activation of downstream immune responses. The induction of GST and other antioxidant-related genes is consistent with the lower MDA accumulation observed in ZJ, suggesting stronger redox regulation and reduced oxidative damage in the resistant cultivar. Similarly, the enhanced expression of defense- and stress-related genes, together with higher POD and PAL activities, indicates that ZJ may activate antioxidant defense and phenylpropanoid-related metabolism more efficiently than NY. In contrast, delayed or weaker induction of these genes in NY may partly explain its higher oxidative damage and susceptibility to *Fof*.

Despite these findings, several limitations of the present study should be acknowledged. First, the experiments were conducted using a single *Fof* isolate; therefore, the extent to which the observed resistance responses are conserved across genetically diverse isolates remains unclear. Second, transcriptome profiling was performed at a limited number of sampling time points and may not fully capture the temporal dynamics of the strawberry response throughout infection. Third, the present analysis mainly focused on DEG identification, GO and KEGG enrichment, and RT-qPCR validation of selected candidate genes. Consequently, the proposed relationships among physiological changes, enriched pathways, and candidate genes are primarily based on associative evidence, and a comprehensive regulatory network was not established. In addition, pathogen biomass, hormone levels, and defense-related metabolites were not directly quantified, and the biological functions of the identified candidate genes have not yet been experimentally validated. Future studies incorporating multiple pathogen isolates, denser temporal sampling, pathogen quantification, metabolite and hormone measurements, and genetic or biochemical validation of candidate genes would help to further substantiate the proposed resistance mechanisms and facilitate their application in strawberry resistance breeding.

## 4 Conclusion

Screening of 64 strawberry accessions identified abundant Fusarium wilt-resistant germplasm, particularly among wild accessions. Comparative analyses of the resistant cultivar ZJ and the susceptible cultivar NY showed that ZJ accumulated more soluble sugars, maintained lower oxidative damage, and exhibited higher POD and PAL activities following inoculation with *Fof*.

Transcriptome analysis revealed rapid and extensive defense-related transcriptional reprogramming in ZJ at 24 h post-inoculation, whereas NY exhibited a delayed response at 120 h. qRT-PCR results supported the RNA-seq expression patterns and prioritized candidate genes associated with pathogen recognition, signal transduction, redox regulation, and protein homeostasis. Collectively, these findings suggest that timely immune activation and coordinated physiological and metabolic responses are closely associated with Fusarium wilt resistance in strawberry. The resistant germplasm and candidate genes identified here provide useful resources for functional validation and resistance breeding.

## Data Availability

Raw RNA-seq transcriptome sequencing data generated in this study were deposited in the NCBI Sequence Read Archive (SRA) under BioProject accession PRJNA1505151 (https://www.ncbi.nlm.nih.gov/sra/PRJNA1505151). The sample accession numbers within this BioProject range from SRX34642485 to SRX34642514. GenBank information of fungal marker genes: the ITS, TEF1, and Fofra sequences correspond to accessions PZ710480, PZ760899, and PZ760900 separately.
